# Gut microbiota depletion minimally affects the daily voluntary wheel running activity and food anticipatory activity in female and male C57BL/6J mice

**DOI:** 10.3389/fphys.2023.1299474

**Published:** 2023-12-01

**Authors:** David E. Ehichioya, S. K. Tahajjul Taufique, Isabel Magaña, Sofia Farah, Yuuki Obata, Shin Yamazaki

**Affiliations:** ^1^ Department of Neuroscience, UT Southwestern Medical Center, Dallas, TX, United States; ^2^ Department of Immunology, UT Southwestern Medical Center, Dallas, TX, United States; ^3^ Peter O’Donnell Jr. Brain Institute, UT Southwestern Medical Center, Dallas, TX, United States

**Keywords:** circadian, antibiotics, restricted feeding, locomotor activity, food-entrainable oscillator, reward, sex differences, gut microbiome

## Abstract

Emerging evidence has highlighted that the gut microbiota plays a critical role in the regulation of various aspects of mammalian physiology and behavior, including circadian rhythms. Circadian rhythms are fundamental behavioral and physiological processes that are governed by circadian pacemakers in the brain. Since mice are nocturnal, voluntary wheel running activity mostly occurs at night. This nocturnal wheel-running activity is driven by the primary circadian pacemaker located in the suprachiasmatic nucleus (SCN). Food anticipatory activity (FAA) is the increased bout of locomotor activity that precedes the scheduled short duration of a daily meal. FAA is controlled by the food-entrainable oscillator (FEO) located outside of the SCN. Several studies have shown that germ-free mice and mice with gut microbiota depletion altered those circadian behavioral rhythms. Therefore, this study was designed to test if the gut microbiota is involved in voluntary wheel running activity and FAA expression. To deplete gut microbiota, C57BL/6J wildtype mice were administered an antibiotic cocktail via their drinking water throughout the experiment. The effect of antibiotic cocktail treatment on wheel running activity rhythm in both female and male mice was not detectable with the sample size in our current study. Then mice were exposed to timed restricted feeding during the day. Both female and male mice treated with antibiotics exhibited normal FAA which was comparable with the FAA observed in the control group. Those results suggest that gut microbiota depletion has minimum effect on both circadian behavioral rhythms controlled by the SCN and FEO respectively. Our result contradicts recently published studies that reported significantly higher FAA levels in germ-free mice compared to their control counterparts and gut microbiota depletion significantly reduced voluntary activity by 50%.

## 1 Introduction

Circadian rhythms are fundamental properties that are controlled by autonomous circadian pacemakers. The primary central circadian pacemaker is located in the suprachiasmatic nucleus (SCN) of the hypothalamus. It is also known that circadian pacemakers exist outside of the SCN (Stephan et al., 1979; Mistlberger, 1994; Davidson, 2009; Honma and Honma, 2009; Pendergast and Yamazaki, 2018). The food-entrainable oscillator (FEO) is one of those extra-SCN pacemakers. Food anticipatory activity (FAA) is a behavioral rhythm that is defined as an increased arousal and locomotor activity that occurs several hours before timed restricted food availability which is controlled by the FEO (Mistlberger, 1994; Stephan, 2002; Davidson, 2009; Silver and Kriegsfeld, 2014; Pendergast and Yamazaki, 2018). One unrevealed nature of the FEO is its time keeping mechanism. It has been shown that the circadian genes are essential for circadian time keeping in the SCN, and circadian oscillators in peripheral tissues are not required for the FEO (Pendergast and Yamazaki, 2018). Despite many attempts at anatomical and genetic ablation approaches, the neural substrate of the FEO remains unclear (Davidson, 2009; Mistlberger, 2011).

Much evidence has indicated the role of the gut microbiota in modulating various aspects of host physiology (Tremaroli and Bäckhed, 2012; Gershon and Margolis, 2021). Multiple studies have shown that the composition of gut microbiota expresses daily changes (Thaiss et al., 2014; Leone et al., 2015; Liang et al., 2015). This rhythm is also affected by timed restricted feeding (Thaiss et al., 2014; Zarrinpar et al., 2014; Zeb et al., 2020; Brooks et al., 2021; Xia et al., 2023). One study showed that human gut bacteria exhibit circadian rhythm *in vitro*, suggesting that gut microbiota has an autonomous circadian oscillator (Paulose et al., 2016). Through bidirectional communication with the brain by means of neural, endocrine, and humoral links, the gut microbiota may be capable of influencing the timing of behavioral and physiological processes. The gut microbiota has been recognized for its role in influencing circadian rhythms and sleep (Brown et al., 1990; Thaiss et al., 2016; Kuang et al., 2019; Szentirmai et al., 2019; Matenchuk et al., 2020; Ogawa et al., 2020). One study suggested that the gut microbiota influences the speed of light entrainment of the circadian pacemaker (Thaiss et al., 2014). Recent studies have demonstrated that gut microbiota influence the robustness of SCN-controlled wheel running rhythm and the FEO-controlled FAA (Dohnalová et al., 2022; Leone et al., 2022). One study showed that germ-free mice exhibit enhanced FAA. In contrast, another study reported that gut microbiota depletion drastically reduced the amplitude of wheel running rhythm. Those studies suggest that gut microbiota differentially regulates circadian rhythms driven by the SCN and the FEO. To test this hypothesis, we depleted the gut microbiota in C57BL/6J mice by administering an antibiotic cocktail via their drinking water throughout the entire experiment and exposed mice to timed restricted feeding during the daytime. Our results show that antibiotics administration did not result in significant changes to either expression of FAA or voluntary wheel running activity rhythm.

## 2 Materials and methods

### 2.1 Animals

**FIGURE 1:**
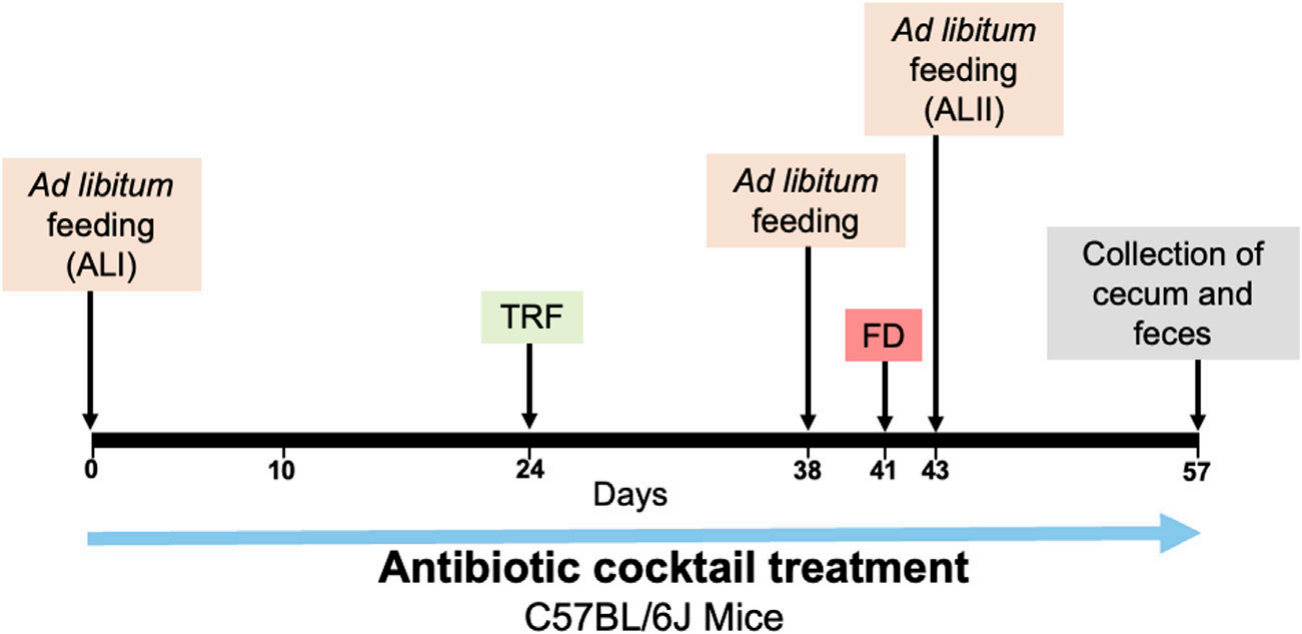
The experimental procedure for microbiota depletion and feeding schedule. The days of beginning of *ad libitum* feeding (ALI, ALII), timed restricted feeding (TRF), food deprivation (FD) and collection of cecum and feces are indicated with the arrows.

Seven weeks old male and female C57BL/6J mice purchased from Jackson Laboratory (stock number 000664) were individually housed in running wheel cages with woodchip bedding (Sani-Chips, PJ Murphy Forest Products) in a 12 h light: 12 h dark condition. Mice were randomly assigned to either the control group (4 males, 5 females) or the antibiotics-treated group (5 males, 5 females). They were initially provided with *ad libitum* access to chow (Teklad Global 18% Protein Rodent Diet 2918; Harlan, Madison, WI, United States) and went through feeding conditions indicated in Figure 1. All mice were in the same light-tight box, handled the same way, and went through each feeding condition at the same time. All experimental procedures were approved by the Institutional Animal Care and Use Committee (IACUC) at UT Southwestern Medical Center (APN 2016-101376-G) and were conducted in accordance with the guidelines of the National Institutes of Health Guide for the Care and Use of Laboratory Animals.

### 2.2 Activity recording

Running wheel cages were placed inside a light-tight ventilated cabinet, with temperature, humidity, and light intensity inside of the cabinet recorded every 10 min using a Chamber Controller software (version 4.104, Actimetrics, Wilmette, IL, United States). The cages were 32.5 cm in length, 14.5 cm in width, and 13 cm in height. The size of the running wheel in the cage is 11 cm in diameter (inside diameter is 10.7 cm). The temperature and humidity inside the cabinet during the experiment were 22.8 ± 2.4 (SD) °C and 40.6 ± 7.9 (SD) %, respectively. Micro-switches mounted to the side of the wheel cages were used to record the numbers of wheel running revolutions every 1 min by ClockLab acquisition system (version 3.604, Actimetrics, Wilmette, IL, United States). The light source inside the cabinet was white LEDs, with an approximate intensity of 100 lux at the cage level during the light period (daytime). The light intensity of the LEDs was controlled by the Chamber Controller software. The actual time of lights on and lights off inside the cabinet was recorded by ClockLab. Zeitgeber time (ZT) 0 is defined as the time of lights on and ZT12 is the time of lights off.

### 2.3 Restricted feeding

As an experimental procedure shown in Figure 1, mice were administered a 4 mM acetic acid solution containing 1 g/L neomycin, 1 g/L ampicillin, 1 g/L metronidazole, and 0.5 g/L vancomycin (Sigma-Aldrich), and 1% (v/w) artificial sweet flavor (Vimto) via their drinking water (Obata et al., 2020). This solution was administered continuously throughout the entire duration of the experiment. The control group received a 4 mM acetic acid solution containing 1% (v/w) artificial sweet flavor (Vimto) alone, also administered via drinking water. All solutions were freshly made and replaced every 7 days.

In the first 3 weeks of the experiment, mice had *ad libitum* access to chow. Starting at ZT12, mice were placed on an overnight fast for a duration of 16 h. Subsequently, starting at ZT4 the next day, we started with 8 h of restricted feeding from ZT4-12 for 2 days, then 6 h of restricted feeding from ZT4-10 for another 2 days. This was followed by 4 h of restricted feeding for a period of 10 days during which food was available from ZT4-8. After the period of timed restricted feeding, mice were then fed *ad libitum* for 3 days. To determine if FAA persists in the absence of food, mice were fasted for 48 h, starting at ZT12. After the period of fasting, the mice were fed *ad libitum* for another 2 weeks.

To validate the efficacy of antibiotics treatment, mice were euthanized by cervical dislocation at the end of the experiment, and the cecum and colon of both control and antibiotics-treated mice were carefully isolated. The wet weight of cecum was measured using a weighing scale (Supplementary Figures S5A–D). Fresh fecal pellets were collected from the colon of both antibiotics-treated and control mice, snap-frozen, and stored at −80°C until DNA isolation for the quantification of bacteria load.

### 2.4 Fecal DNA extraction and fecal microbiota composition analysis

DNA from fecal pellets was isolated using the ZymoBIOMICS DNA/RNA MiniPrep Kit (Zymo, R2002) according to the manufacturer’s protocol. Quality and concentration of the extracted DNA were evaluated using the NanoDrop 2000c Spectrophotometer (Thermo Fisher). Extracted fecal DNA samples were shipped to Transnetyx Microbiome (Cordova, TN) for library preparation and whole-genome sequencing. The Transnetyx platform employed a minimum read depth of 2 million paired-end reads for sequencing and provided a species and strain level taxonomic resolution on each sample. Sequenced data were uploaded automatically onto One Codex (San Franciso, CA) microbiome analysis cloud computing website (https://www.onecodex.com) and were analyzed against the One Codex database which consists of approximately 127 thousand whole microbial reference genomes. One Codex utilizes NCBI for taxonomy classification. In cases where a microbe does not have an Order, Family, or Genus assigned, it is denoted as “No Order”, “No Family,” or “No Genus” in that taxonomic rank. We selected to display the top 15 abundant taxa. Microbes in the samples that are not in the top 15, including the non-bacterial microbes, are categorized as “Other”. All sequence data in the current study are accessible from this link (https://app.onecodex.com/projects/tyx_6ba12d872a8).

### 2.5 Calculation of the total number of 16S rRNA gene copies in fecal DNA

DNA from DH5α *Escherichia coli* (Fisher, FEREC0111) was used to generate a standard curve for estimating the total number of 16S rRNA gene copies/mg feces as previously described (Lynn et al., 2022). Colony-forming units (CFU/mL) of cultured *E. coli* in LB broth (Sigma-Aldrich, L3522) were determined by counting the number of colonies on LB agar plates (Sigma-Aldrich, L3147). qRT-PCR was performed using the Applied Biosystems QuantStudio 7 Flex Real-Time PCR System. The standard curve graph was generated by plotting the log10 (DNA copies/ml) vs. the cycle threshold values of the amplified *E coli* DNA standards. The cycle threshold values of each fecal DNA sample were plotted on the standard curve to determine the total number of 16S rRNA gene copies/mg feces.

### 2.6 Data analysis

ClockLab analysis software (version 3.604, Actimetrics) was used to generate double-plotted actograms (10-min bins, using the scale plot of 0–100). Group average activity profiles were generated by averaging daily locomotor activity in 10-min bins during either 7 days of *ad libitum* feeding, first 4 days of timed restricted feeding (8 h–6 h), last 7 days of 4 h timed restricted feeding, or 7 days in subsequent *ad libitum* feeding from individual mice. Then group averaged mean of 24-h activity profiles were plotted relative to the light-dark cycle. FAA was quantified by measuring the total number of wheel revolutions in the 3 h period preceding food availability (ZT1 to ZT4). Daily changes in FAA (Figures 3A,B) or FAA as the proportion of daily activity (Supplementary Figure S2) were analyzed. FAA duration was determined as follows. We set a minimum activity threshold with an amplitude of 100 counts/10 min. Then, we calculated the time between wheel revolutions exceeding the set threshold within the 3 h window prior to food availability. Also, we compared daily water intake over the initial 22 days, food intake (days 24–35), and body weight measured on days 1, 23, and 37 between the control and antibiotics-treated groups.

### 2.7 Statistical analysis

A comparison between control and antibiotics-treated groups was done with Mann Whitney U-test. For a two-group comparison with time, a two-way repeated measures ANOVA was used. When the statistical difference was found, Tukey’s *post hoc* multiple comparison test was used. All statistical analyses were carried out using GraphPad Prism 9.0 software (GraphPad Software Inc., San Diego, CA, United States). A significance level of *p* < 0.05 was used as the criterion for statistical significance.

## 3 Results

### 3.1 Mice administered antibiotics showed robust FAA

**FIGURE 2:**
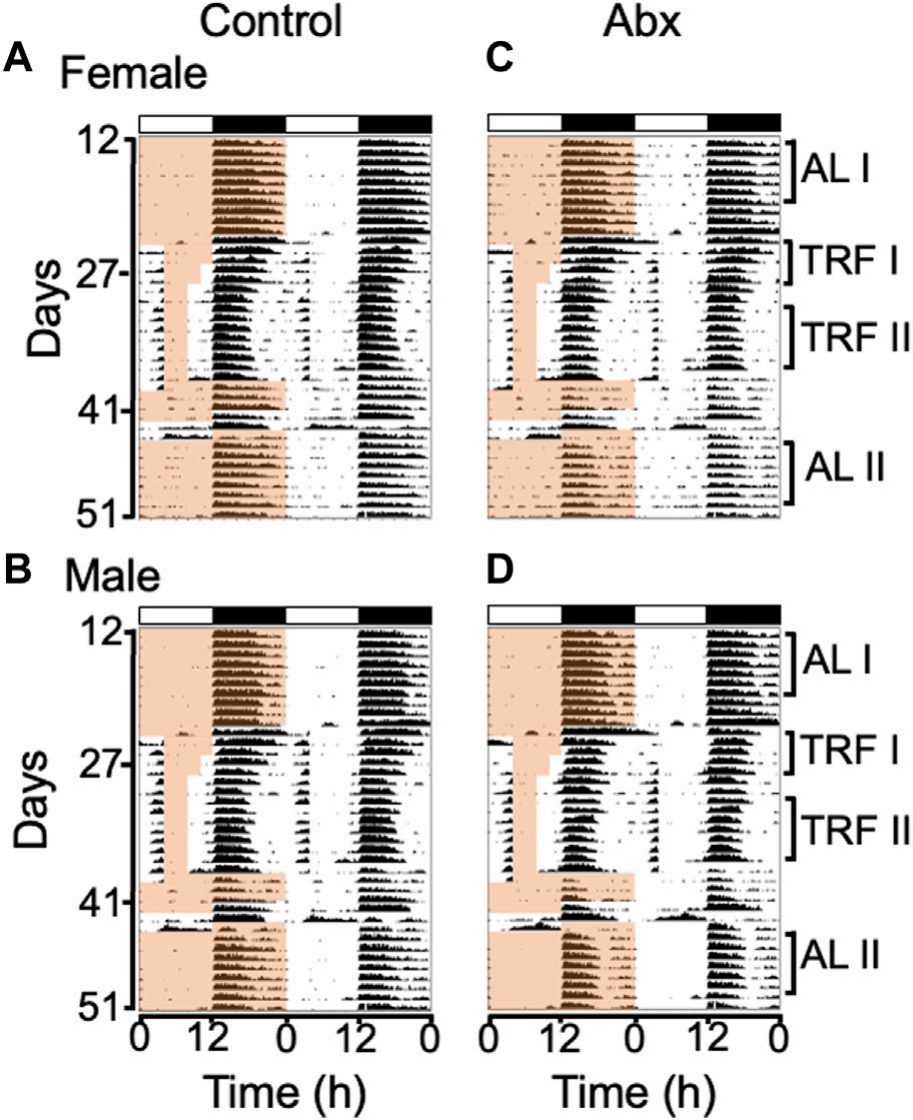
Group average double-plotted actograms of antibiotics-treated and control mice. Group average actograms of control females **(A)**, control males **(B)**, antibiotics-treated females **(C)**, antibiotics-treated males **(D)** were plotted with 10 min bin and scale format (0–100 counts/min). The light and dark cycle is indicated with white and black bars on top of each actogram. Time of food availability is indicated with the light orange shading only on the left half of each actogram. ALI (*ad libitum* before RF*)*, TRFI (first 4 days of timed restricted feeding), TRFII (last 7 days of timed restricted feeding) and ALII (*ad libitum* after RF) labeled on right side of the actograms indicates the days used to generate the daily activity profiles for before, during and after timed restricted feeding shown in Figure 4. Individual actograms of all mice are shown in Supplementary Figure S1.

**FIGURE 3:**
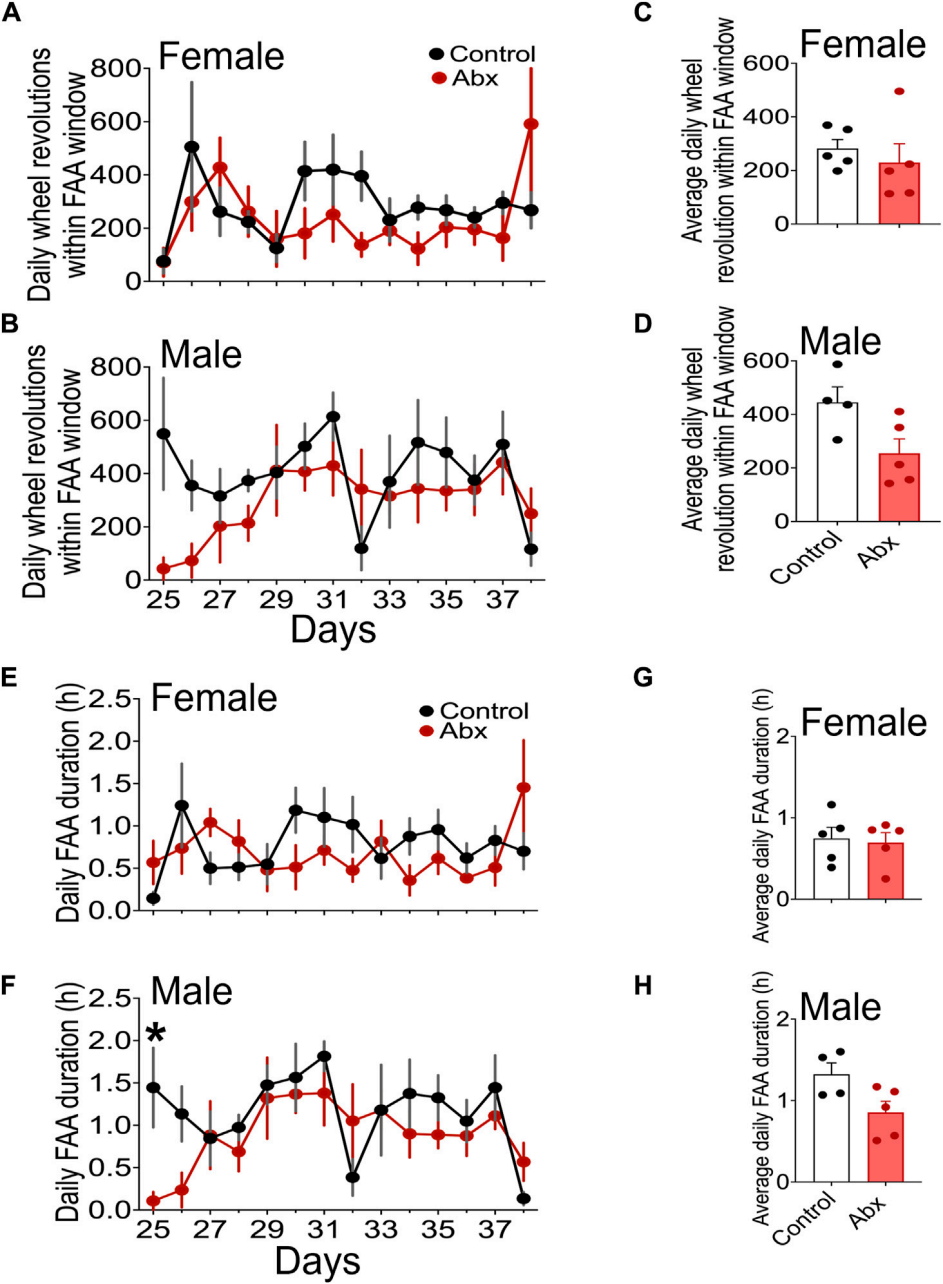
Quantitative analysis of FAA. Daily changes of wheel running revolution in FAA windows (mean ± SEM) in females **(A)** and males **(B)** were presented. Steady state FAA, wheel revolution in FAA windows of the last 7 days of 4 h restricted feeding schedule were shown in **(C)** (females) and **(D)** (males). Daily changes of FAA duration in females **(E)** and males **(F)** and FAA duration in the last 7 days of 4 h restricted feeding in females **(G)** and males **(H)** were shown. Plots represent mean ± SEM. Two-way repeated measures ANOVA detected a significance only in **(F)** * represents *p* < 0.05 by Tukey’s *post hoc* multiple comparison test.

**FIGURE 4:**
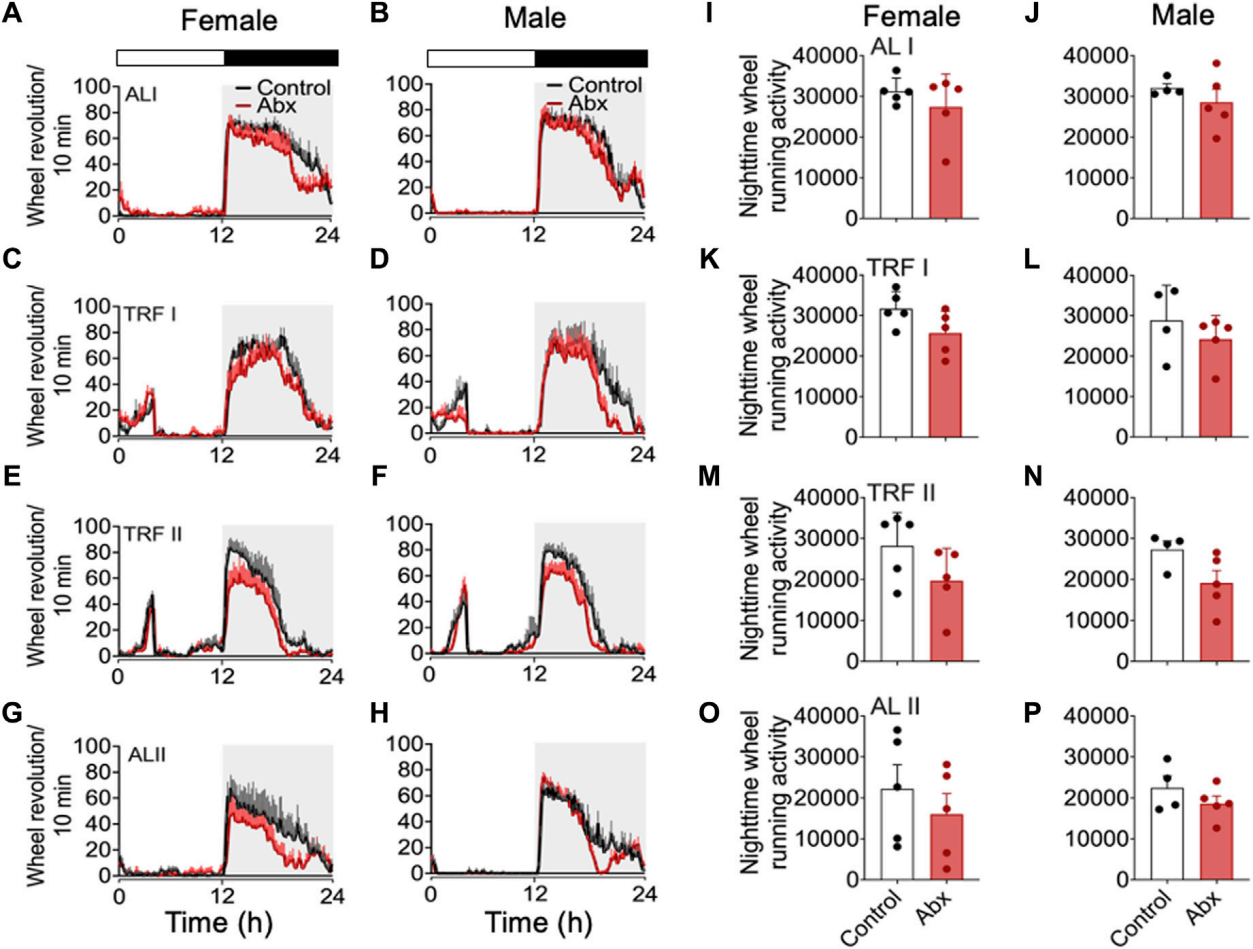
Group average activity profiles and nighttime activity before, during and after timed restricted feeding. Group average activity profiles in females **(A)**: ALI, **(C)** TRFI, **(E)** TRFII, **(G)** ALII) and males **(B)**: ALI, **(D)** TRFI, **(F)** TRFII, **(H)** ALII) were generated during the days indicated in Figure 2. Averaged total number of wheel revolution at night were plotted in **(I–P)** (**I**: female ALI, **(J)** male ALI, **(K)** female RFI, **(L)**: male RFI, **(M)**: female TRFII, **(N)**: male TRFII, **(O)**: female ALII, **(P)**: male ALII). Each plot represents mean ± SEM.

We treated female and male C57BL/6J mice with an antibiotic cocktail, then performed daytime timed restricted feeding (Figure 1). Similar to control mice, antibiotics-treated mice during timed restricted feeding exhibited robust FAA, the daytime wheel-running activity starting 2-3 h before the time of food availability (Figures 2A–D: TRF; Actograms for all individual mice are provided in Supplementary Figure S1). We quantified the robustness of FAA by measuring wheel revolutions during the 3 h period that precedes food availability (FAA window). Because two-way repeated measures ANOVA failed to detect a significant difference between control and antibiotics-treated groups in both females and males (Figures 3A,B), we didn’t run a *post hoc* analysis. However, the mean of running wheel revolutions during the FAA window in antibiotics-treated female and male mice was lower compared with that in control females and males (Figures 3A,B). There was very weak FAA in antibiotics-treated male mice the first 4 days of restricted feeding (Figures 3B, 4D) suggesting that antibiotics-treated male mice developed FAA slower compared to control males. However, there was no statistical difference in mean wheel running activity in FAA window during the last 7 days of 4 h restricted feeding between antibiotics-treated and control groups in both males and females (Figures 3C,D). We also evaluated FAA as the proportion of daily total activity (Supplementary Figure S2). Although a two-way repeated measure ANOVA didn’t detect significance in both females and males, FAA (% activity) in antibiotics-treated males showed a lower number compared with that in control males during first 4 days of timed restricted feeding. We next compared duration of FAA. Although a two-way repeated measures ANOVA didn’t detect a significant difference in FAA duration between control and antibiotics-treated groups in females, a two-way repeated measures ANOVA detected significance of that in males (Figures 3E,F). *Post hoc* analysis detects the difference between antibiotics-treated males and control males at only the first day of restricted feeding. There was no statistical difference in overall duration of FAA at the last 7 days of restricted feeding between antibiotics-treated mice and the control group in either males or females (Figures 3E–H).

To test the effect of microbiota depletion on autonomous rhythmicity of the FEO, we measured FAA during 48 h food deprivation. All mice exhibited FAA coinciding with the previous feeding schedule during 48 h food deprivation (Figure 2, Supplementary Figure S1). This strongly suggests that microbiota is not essential for rhythm generation in the FEO.

### 3.2 Antibiotics administration did not significantly alter nocturnal voluntary wheel running activity

To evaluate the SCN-controlled wheel running activity during antibiotics treatment, we generated 24-h wheel running activity profiles (Figure 4). 7 days of group average 24-h activity profiles at 4 different feeding conditions, during baseline *ad libitum* feeding (days 13–19, represented in Figure 2 as ALI), first 4 days of restricted feeding (days 24–27 represented in Figure 2 as TRFI), last 7 days of restricted feeding (days 31–37 represented in Figure 2 as TRFII), and first 7 days of the subsequent *ad libitum* feeding (days 45–51 represented in Figure 2 as ALII) were analyzed. We also quantified total number of wheel revolutions at night during the respective 4-day or 7-day periods mentioned above. Although a two-way repeated measures ANOVA didn’t pick up significance between antibiotics and control groups, the group mean of wheel running activity was always slightly lower in antibiotics-treated mice compared to that in the control group (Figures 4A–H). There was no statistical difference in the total number of wheel revolutions at night between antibiotics-treated mice and control during any of the feeding schedules (Figures 4I–P). To make a direct comparison with a published study (Dohnalová et al., 2022), we also analyzed distance traveled during the last 3 days of *ad libitum* feeding of control mice (Supplementary Figure S3). The duration of antibiotic administration in this analysis (20-22 days) is comparable with the study. The average distance of travel in control females and males was very similar to that in the published study. Antibiotic-treated females and males showed a slightly reduced distance of travel compared to controls. However, the total distance of travel per day between control and antibiotic-treated females and males was not statistically different. Although the direction of gut microbiota depletion in our study is consistent with published data, our data contradicts the published work which showed that antibiotics-treated mice show ∼50% reduction of activity (Dohnalová et al., 2022).

### 3.3 Effect of antibiotics administration on water and food intake

To assess the potential impact of the antibiotics treatment on fluid consumption, fluid intake was monitored for the initial 22 days of the experiment. There was no difference in fluid consumption between antibiotics-treated mice and the control group (Supplementary Figures S4A,B). However, during timed restricted feeding, male mice administered antibiotics exhibited reduced food intake compared to their control male counterparts, while there was no difference in food consumption between antibiotics-treated females and control females (Supplementary Figures S4C,D). Despite the difference in food intake between the male groups, body weight remained unchanged between male and female antibiotics-treated mice and their control groups (Supplementary Figures S4E,F).

### 3.4 Validation of antibiotics treatment

**FIGURE 5:**
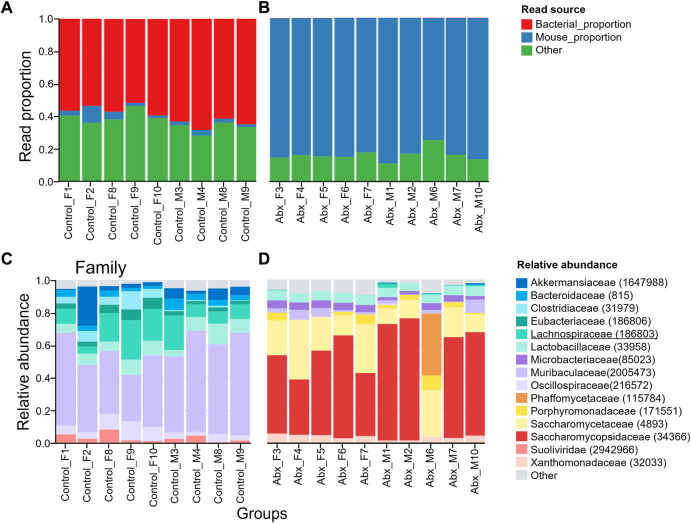
Relative abundances of microbiota taxa from control and antibiotic-treated mice. Plot representing bacterial load in control **(A)** and antibiotics-treated **(B)** mice. Antibiotics depleted majority of gut microbiota. The “Mouse proportion” represents the number of reads that map to the One Codex database mouse genome divided by the total number of reads, while the “Bacterial proportion” represents the number of reads that map to bacteria at the taxonomic rank divided by the total number of reads. The “Other” category includes any reads classified as non-bacterial microorganisms. Taxonomic analysis showing gut microbiota population shifts at Family level. Relative abundance of top 15 families of microbial taxa in control **(C)**, and antibiotics-treated **(D)** are shown. *Lachnospiraceae* (indicated as green) was present in all control mice but depleted in antibiotics treated mice. *Erysipelotrichaceae* was not detected in both control and antibiotics-treated mice. Top 15 orders and genus were shown in Supplementary Figure S5A–D. The number in the brackets represents NCBI taxonomic ID.

To confirm the efficacy of antibiotics treatment in our study, we measured the wet weight of cecum and evaluated changes in bacteria load at the end of the experiment. The increased wet weight of cecum following antibiotics treatment provides evidence of the effectiveness of our antibiotics treatment protocol (Supplementary Figures S5A–D). However, there was detectable 16S rRNA gene copy number by Quantitative PCR (Supplementary Figures S5E,F). This indicates the antibiotics treatment successfully depleted majority of gut microbiota, but microbiota that are resistant to neomycin, ampicillin, metronidazole, and vancomycin were present in gut. Transnetyx sequencing showed a drastic reduction in reads of bacterial proportion in all antibiotics-treated mice (Figures 5A,B).

### 3.5 *Erysipelotrichaceae* and *Lachnospiraceae* are depleted in antibiotic-treated mice gut

We further analyzed fecal microbiota composition with the Transnetyx platform. As consistent with a previously published study (Dohnalová et al., 2022), microbiota in family *Lachnospiraceae* were depleted in the antibiotics-treated mice (Figures 5C,D). Unlike the published study, family *Erysipelotrichaceae* is negligibly expressed in our mice, with read values of 457.7 ± 183.9 in our control group and 0.2 ± 0.6 in the antibiotic-treated group. Among those families, *Eubacterium rectale* and *Coprococcus eutactus* were identified to play significant roles in enhancing the amount of wheel running activity in published study. Therefore, we specifically looked at those two species of gut microbiota in our mice. Transnetyx sequencing didn’t detect both *C. eutactus* and *E. rectale* in our mice. If those species are responsible for enhancing voluntary wheel running activities, our mice should exhibit low levels of wheel running activity. However, the distance of travel in our control mice was nearly identical of that in published work with the presence of those two species of bacteria (Dohnalová et al., 2022). The genus *Coprococcus* is negligibly present in our mice (the read value 708.9 ± 364 in control group, 0.3 ± 0.6 in antibiotics-treated group). The genus *Eubacterium* was present in control mice and depleted in antibiotics-treated mice (Supplementary Figures S6C–D). Depletion of *Eubacterium* was less pronounced in one antibiotics-treated individual (M1). However, the distance of travel of M1 was similar to other antibiotics-treated mice (Supplementary Figure S3). Altogether, our data suggests that *Erysipelotrichaceae* and *Lachnospiraceae* families have minimum effects on voluntary wheel running activity.

## 4 Discussion

The gut microbiota has gained significant recognition for its role in regulating various behavioral and physiological processes, including metabolism and feeding behavior (Tremaroli and Bäckhed, 2012; Frost et al., 2014; Gershon and Margolis, 2021). Recent studies have demonstrated that gut microbiota regulates sleep and circadian rhythms (Szentirmai et al., 2019; Matenchuk et al., 2020; Ogawa et al., 2020). Leone and colleagues demonstrated that germ-free mice exhibit stronger FEO-controlled FAA compared with that in controls. In contrast, Dohnalová and colleagues have shown that the SCN-controlled nocturnal wheel-running activity was ∼50% reduced by antibiotics treatment. Therefore, we speculated that gut microbes regulate SCN-controlled nocturnal activity and FEO-controlled FAA differently. In our current study, mice treated with an antibiotic cocktail showed clear FAA similar to the control mice during timed restricted feeding and food deprivation. Therefore, the gut microbiota is not essential for FAA expression. A previous study (Leone et al., 2022) observed FAA in germ-free mice was significantly higher compared to their control group. This is different from our current report where we observed no statistical difference in FAA between antibiotics-treated mice and their control groups with a smaller sample size compared with previous study. Although a two-way repeated measures ANOVA failed to detect statistical difference, the number of wheel revolutions within the 3 h FAA window is consistently lower in antibiotics-treated mice compared to control mice. Consistent with previous studies (Li et al., 2015; Michalik et al., 2015; Aguayo et al., 2018), male mice develop FAA faster than female mice. There was only a statistical difference in the FAA duration between antibiotics-treated males and control males on the second day of restricted feeding. This suggests that the effect of gut microbiota had a minimal impact on FAA development and the statistical difference was barely detectable only in rapidly developing FAA in males. It is well-known that antibiotics do not completely deplete all gut microbiota (Kennedy at el., 2018). This may contribute to the different outcomes in the two studies. However, a change in FAA in our study is in the opposite direction from what Leone et al. (2022) reported. It is still possible that common microbiota present in our antibiotic-treated mice and SPF control mice of the Leone et al. study attenuated the FAA. Another difference between the two studies is the photoperiod mice were raised in. We obtained the mice from the Jackson Laboratory. The Jackson Laboratory uses LD 14:10 light conditions for their mice facility. Although we conducted the experiment with standard LD 12:12 light conditions, those mice were raised under the long day condition. It has been demonstrated that long-day exposure induces neurotransmitter switch somatostatin to dopamine in the hypothalamus (Dulicis et al., 2013). It is possible that mice raised long-day may have elevated dopamine and maximized robustness of FAA.

Our data also showed that with antibiotics administration, daily locomotor activity behavior was minimally affected both during *ad libitum* and timed restricted feeding. While we observed that the mean nighttime locomotor activity in the antibiotics-treated group was always slightly lower compared with that in control, a two-way repeated measures ANOVA failed to detect any statistical difference. The effect of antibiotics treatment on SCN-controlled nighttime locomotor activity reported in the study of Dohnalová et al. (2022) is approximately 50% reduced in antibiotics-treated mice. In their study, C57BL/6J mice (obtained from the Jackson Laboratory) were treated with 6 different antibiotics, including imipenem and ciprofloxacin which were not in our antibiotic cocktail. By single antibiotic treatment and single specie colonization in germ-free mice, the authors suggested that *E. rectale* and *C. eutactus* are likely species to enhance voluntary wheel running activity in their mice. Fecal microbiota composition analysis in our study showed both species are absent in both control and antibiotics-treated mice. Despite the absence of two species of bacteria, the distance of travel in our control mice is nearly identical to that of Dohnalová *et al* study. Antibiotics-treated mice in our study had no significant changes in wheel running activity, which contrasts with Dohnalová et al. study showing ∼50% reduction. It is possible the bacteria species present in our antibiotics-treated mice and absent in antibiotics-treated mice in Dohnalová et al. study enhanced voluntary activity in our mice. However, the study, Leon et al., in which germ-free mice exhibit comparable running wheel activity with control SFP mice, suggests that this possibility is less likely.

A limitation of our current study is the small sample size. The sample size is enough to conclude the presence of normal FAA during timed restricted feeding in antibiotics-treated mice. However, the sample size of our current study is not large enough to provide strong statistical power to test if antibiotics treatment affected the development and robustness of FAA controlled by the FEO, as well as the robustness of nocturnal activity controlled by the SCN. The trends seen by our current study may motivate researchers to conduct studies with larger sample sizes. Another limitation of our study is that we evaluated the efficiency of bacterial depletion using fecal stool samples collected at the end of the experiment. Although previous studies demonstrated that 3 weeks of antibiotic administration is enough to deplete the gut microbiota (Kennedy et al., 2018; Dohnalová et al., 2022; Tan et al., 2022), the effect of antibiotic treatment on bacterial abundance and diversity at the time when we measured FAA (days 24–38) may be smaller than that at the end of the experiment (day 57).

In conclusion, our data suggest that the gut microbiota is not a necessary component for FAA in C57BL/6J female and male mice. We demonstrated that gut microbiota depletion had minimal influence on FAA expression as well as SCN-controlled nocturnal activity. The trend in our study suggests there is a possible small enhancement effect of gut microbiota on both activities controlled by the SCN and the FEO. This is in line with Dohnalová et al. (2022)-though the effect is much smaller in our study- but contradicts with Leone et al. (2022).

## Data Availability

The datasets presented in this study can be found in online repositories. The names of the repository/repositories and accession number(s) can be found below: https://app.onecodex.com/projects/tyx_6ba12d872a8.
