# Supplementary material for: Gut microbiota depletion minimally affects the daily voluntary wheel running activity and food anticipatory activity in female and male C57BL/6J mice
Source: Front Physiol. 2023 Dec 1;14:1299474. doi: 10.3389/fphys.2023.1299474 (PMC10722266; doi:10.3389/fphys.2023.1299474)
Supplement: Supplementary file 1 [file Image1.pdf]

Supplementary Material

Gut microbiota depletion minimally affects the daily voluntary wheel running activity and food anticipatory activity in female and male C57BL/6J mice.

David E. Ehichioya, S K Tahajjul Taufique, Isabel Magaña, Sofia Farah, Yuuki Obata, and Shin Yamazaki*

*** Correspondence:**

Shin Yamazaki: shin.yamazaki@utsouthwestern.edu

Supplementary Figures

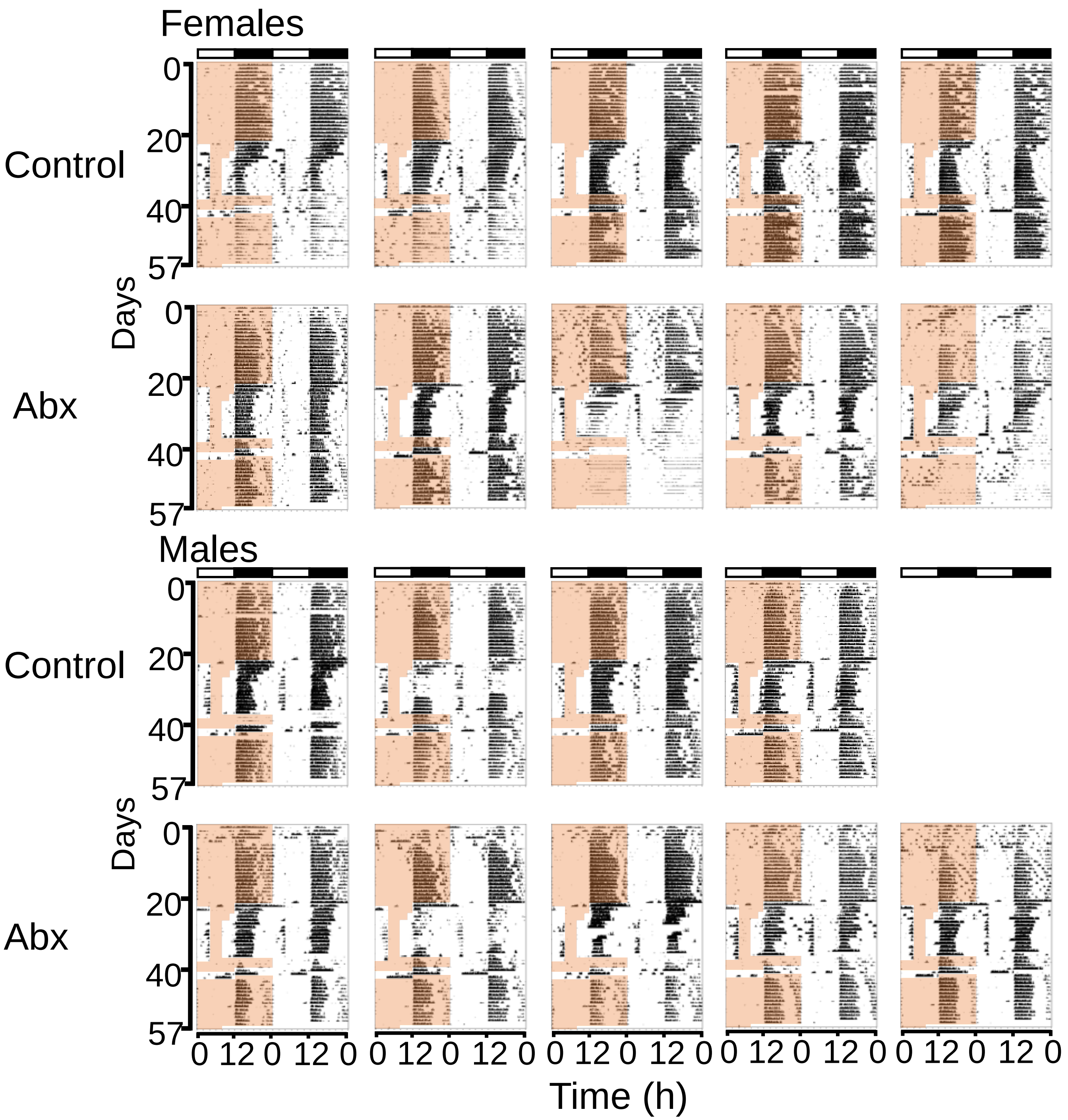

Supplementary Figure S1: Individual double plotted actograms for wheel running activity of all C57BL/6J male and female antibiotics (Abx)-treated and control mice.

The light (day) and night (dark) periods are indicated with white and black bars respectively on top of each actogram. The time of food availability is indicated with the light orange shading on the left half of each double-plotted actogram. Ten-minute bins and a scale format of 0 - 100 were used for all plots.

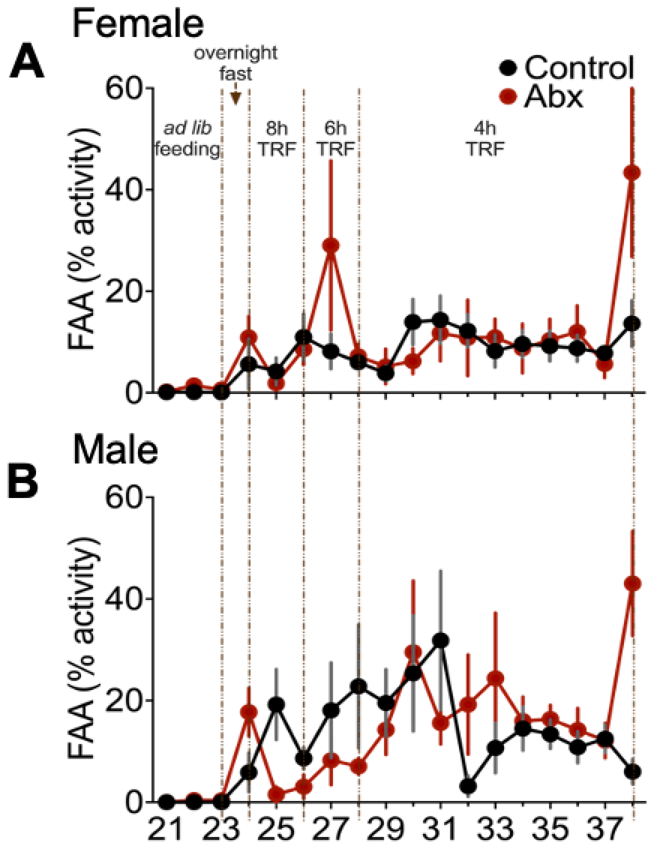

Supplementary Figure S2: Development of FAA over the course of timed restricted feeding.

Total activity during 3 h of FAA windows was quantified as the proportion of daily activity. Changes in daily FAA were plotted from 3 days before TRF (baseline) until the last day of TRF.

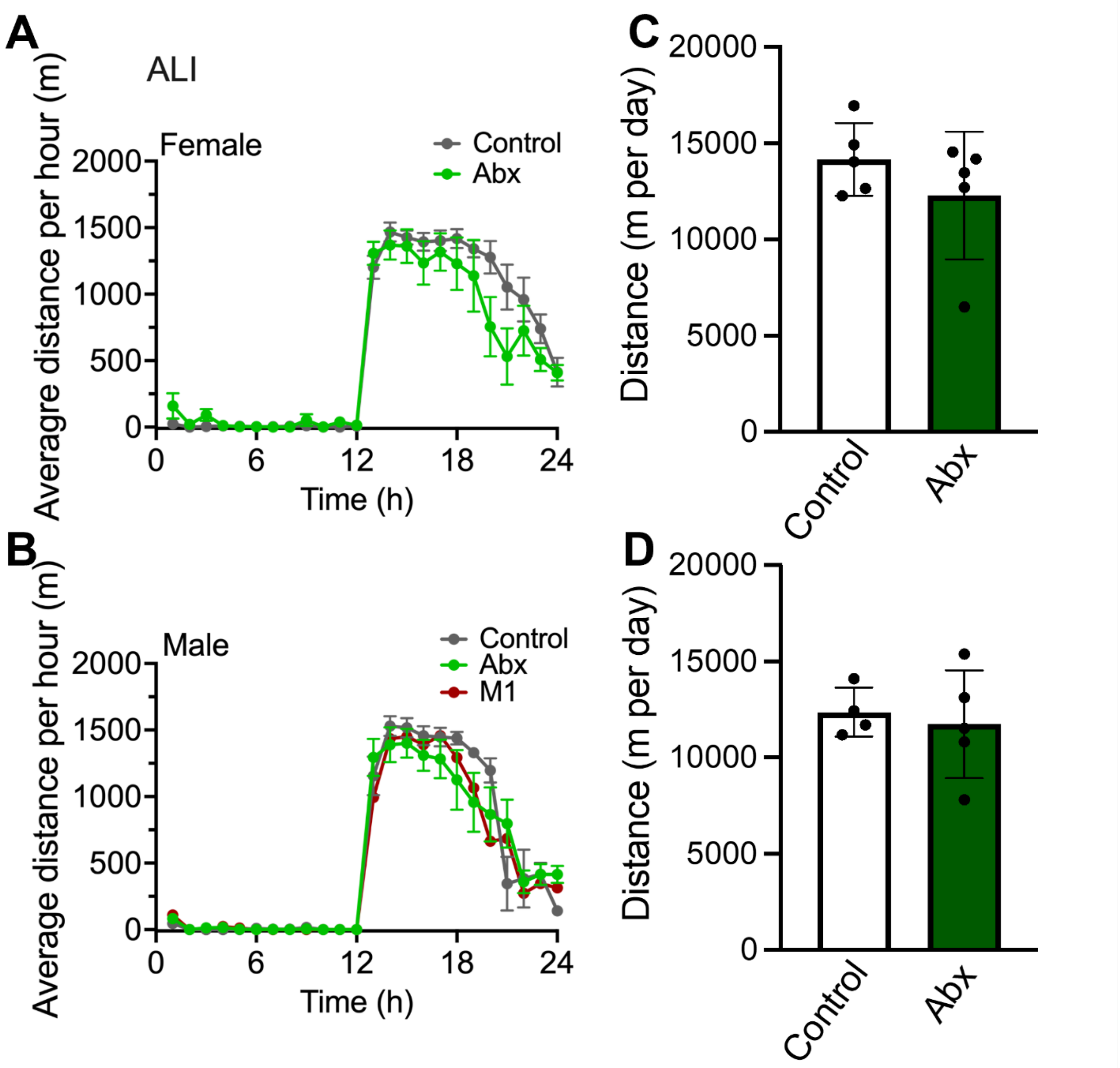

Supplementary Figure S3: Group average distance traveled during antibiotics treatment.

Group average 24 h profile of distance traveled in females (**A**) and males (**B**) were calculated during the last three days of *ad libitum* feeding before timed restricted feeding. The total distance traveled during night is shown in **(C)** for females and **(D)** for males. Distance traveled was determined by multiplying the number of running wheel revolutions by the wheel's circumference. Each plot shows the mean ± SEM.

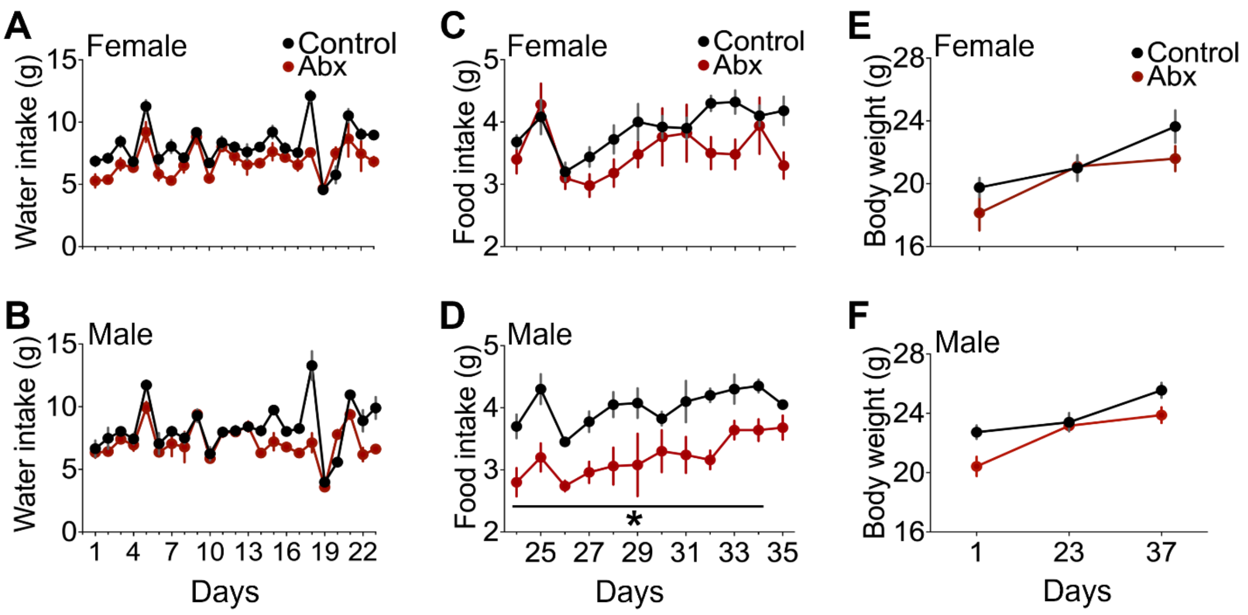

Supplementary Figure S4: Daily fluid and food intake and body weight during antibiotics treatment.

Daily water intake in females **(A)** and males **(B)** in the initial 22 days of the experiment, daily food intake in females **(C)** and males **(D)** during timed restricted feeding (day 24 to day 35) are shown. Body weight was measured on days 1, 23, and 37 (**E**: females, **F**: males). The mean ± SEM is shown. *p< 0.05.

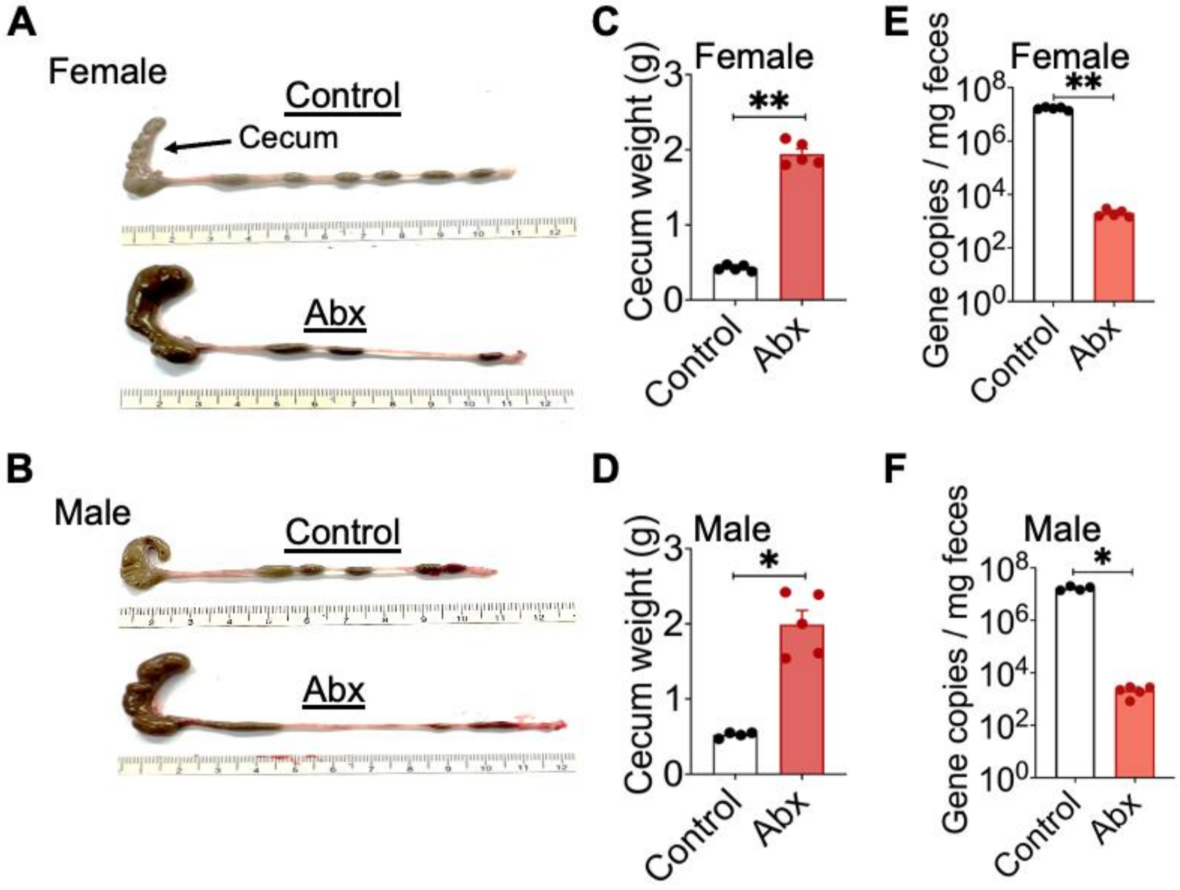

Supplementary Figure S5: **Antibiotic treatment resulted in increased cecal size.**

Representative images of cecum and colon obtained from females **(A)** and males **(B)**. The average wet weight of cecum is shown in **C-D** (**C**: females, **D**: males). The average 16 rRNA gene copies per mg feces measured by qPCR are shown in **E-F** (**E**: females, **F**: males). Each plot represents mean ± SEM. *$p < 0.05$, **$p < 0.01$.

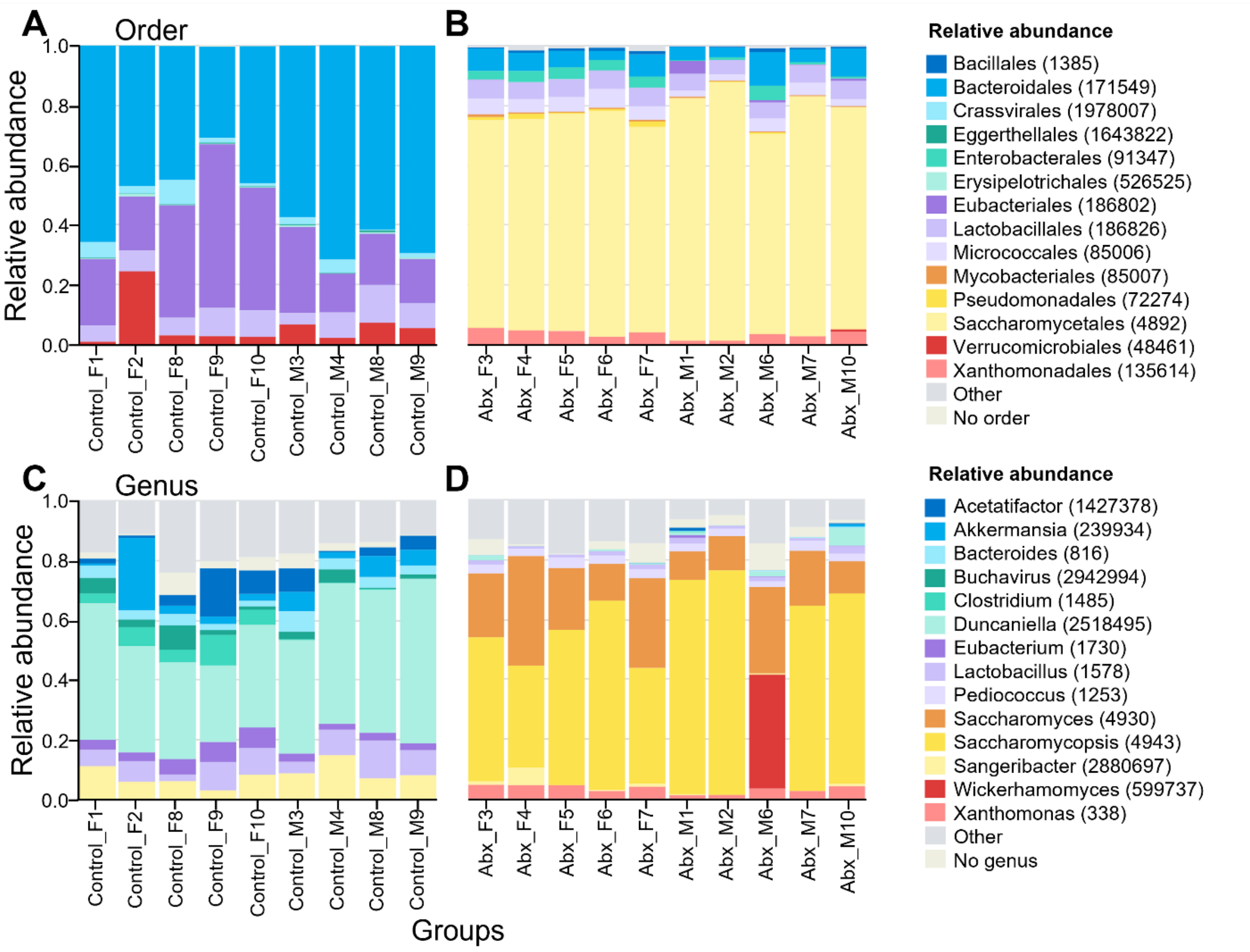

Supplementary Figure S6: **Fecal microbiota composition in control and antibiotic treated mice.**

Taxonomic analysis showing the population of gut microbiota at the order and genus levels. Relative abundance plot of the top 15 orders of microbial taxa is shown in control **(A)** and antibiotics-treated **(B)**. *Erysipelotrichales* (indicated as light teal color) is present in control but completely depleted in antibiotics-treated mice. Relative abundance plot of top 15 genera of microbial taxa in control **(C)** and antibiotics-treated **(D)** are shown. *Eubacterium* (indicated as dark purple) is depleted in most antibiotics-treated mice. The number in the brackets represents NCBI taxonomic ID.
